# Reply: Dear Editor-in-Chief Iranian J Parasitol

**Published:** 2012

**Authors:** Mahdi Fakhar

**Affiliations:** Department of Parasitology and Mycology, School of Medicine, Mazandaran University of Medical Sciences, Sari, Iran

Many thanks for valuable comment of one of our colleagues concerning to our paper entitled: **“Molecular and Seroepidemiological Survey of Visceral Leishmaniasis among Humans and Domestic Dogs in Mazandaran Province, North of Iran”** recently published in Iranian Journal of Parasitology (Volume 6 (4), Oct-Dec 2011).

As you know this is an international journal which is indexed and abstracted by Thompson/ISI and recently in PubMed Central and also in other databases, thus, we must use term forms of the Medical Subject headings in titles as well as in key words for indexing purposes in the international databases. Since, Semeskandeh and Kiakola districts are not appropriate words for indexing in the world databases and they are unfamiliar for overseas readers so, these districts have been completely described in the Materials and Methods section in the last paragraph of Page 52. Moreover, in our paper we have mentioned all previous studies which were published even through in proceeding of congress. We could find three major citations about the earlier VL studies in Mazandaran Province that were mentioned in introduction section including references 13, 14 and 17.

**Mahdi Fakhar (PhD)**

**Corresponding Author**

**Assistant Professor of Medical Parasitology**

**Department of Parasitology and Mycology, School of Medicine,**

**Mazandaran University of Medical Sciences,**

**Sari, Iran**

**Email:** mahdif53@yahoo.com

